# High B3GALT5 expression confers poor clinical outcome and contributes to tumor progression and metastasis in breast cancer

**DOI:** 10.1186/s13058-020-01381-9

**Published:** 2021-01-07

**Authors:** Yu-Mei Liao, Ya-Hui Wang, Jung-Tung Hung, Yu-Ju Lin, Yen-Lin Huang, Guo-Shiou Liao, Ya-Ling Hsu, Jen-Chien Wu, Alice L. Yu

**Affiliations:** 1 Institute of Stem Cell and Translational Cancer Research, Chang Gung Memorial Hospital at Linkou, Taoyuan, 333 Taiwan; 2 Ph.D. Program in Translational Medicine, Kaohsiung Medical University, Kaohsiung, and Academia Sinica, Taipei, 115 Taiwan; 3 Division of Hematology and Oncology, Department of Pediatrics, Kaohsiung Medical University Hospital, Kaohsiung, 807 Taiwan; 4 Department of Anatomic Pathology, Chang Gung Memorial Hospital at Linkou, Taoyuan, 333 Taiwan; 5 Division of General Surgery, Department of Surgery, Tri-Service General Hospital, National Defense Medical Center, Taipei, 114 Taiwan; 6 Graduate Institute of Medicine, College of Medicine, Kaohsiung Medical University, Kaohsiung, 807 Taiwan; 7 Department of Pediatrics, University of California in San Diego, San Diego, USA; 8 Genomics Research Center, Academia Sinica, Taipei, 115 Taiwan

**Keywords:** B3GALT5, Breast cancer, EMT, Metastasis, Clinical outcome

## Abstract

**Background:**

Existence of breast cancer stem cells (BCSCs) is implicated in disease relapse, metastasis, and resistance of treatment. β1,3-Galactosyltransferase 5 (B3GALT5) has been shown to be a pro-survival marker for BCSCs. However, little is known about the prognostic significance of B3GALT5 in breast cancer.

**Methods:**

Paired tissues (tumor part and adjacent non-tumor part) from a cohort of 202 women with breast cancer were used to determine the expression levels of *B3GALT5* mRNA by qRT-PCR. Kaplan–Meier and multivariable Cox proportional hazard models were used to assess survival differences in terms of relapse-free survival (RFS) and overall survival (OS). Both breast cancer cells and cancer stem cells (BCSCs) were used to see the in vitro effects of knockdown or overexpression of B3GALT5 on cell migration, invasion, and epithelial-to-mesenchymal transition (EMT). A patient-derived xenograft (PDX) model was used to see the in vivo effects of knockdown of B3GALT5 in BCSCs on tumor growth and metastasis.

**Results:**

Higher expression of *B3GALT5* in 202 breast cancer tissues, especially in adjacent non-tumor tissue, correlated with poor clinical outcomes including shorter OS and RFS in all patients, especially those with early stage breast cancer. In vitro studies showed B3GALT5 could enhance cell migration, invasion, mammosphere formation, and EMT. Of note, B3GALT5 upregulated the expression of β-catenin and EMT activator zinc finger E-box binding homeobox 1 (ZEB1) pathway in BCSCs. In vivo studies showed B3GALT5 expression in BCSCs is critical for not only tumor growth but also lymph node and lung metastasis in PDX mice.

**Conclusion:**

Our results demonstrated the value of B3GALT5 as a prognostic marker of breast cancer, especially among the early stage patients, and its crucial roles in regulating EMT, cell migration, and stemness thereby promoting breast cancer progression.

## Introduction

Breast cancer is the most common cancer among women worldwide and the second leading cause of cancer-related mortality in women [1]. Despite the improvements in multidisciplinary therapy have led to increasing chances for cure in ~ 70–80% of patients with early stage breast cancer, metastatic disease is not considered curable [2]. Only 5–10% of cases are genetically predisposed [3]; the pathogenetic mechanisms of most breast cancer remain largely unknown. Epithelial-to-mesenchymal transition (EMT) and the presence of breast cancer stem cells (BCSCs) are two of the features associated with tumor aggressiveness, treatment resistance, metastasis, and poor prognosis [4–6]. Targeting EMT- and cancer stem cells (CSCs) might be a very desirable addition to the existing therapeutic armamentarium for breast cancer.

Recently, targeting therapies directed against kinases have emerged as the mainstay of cancer treatment. Actually, aberrant glycans or glycosylation is a very common occurrence in cancer, which has yet to be exploited as target for cancer therapy except for GD2. Glycoconjugates, including glycoprotein, glycolipid, and proteoglycan, play important roles in EMT and maintenance of CSCs [7, 8]. β1,3-Galactosyltransferase 5 (B3GALT5) is an enzyme of type I chain carbohydrate synthase in mammals. The generation of glycolipid stage-specific embryonic antigen-3 (SSEA-3) is catalyzed by B3GALT5 [9]. It has been reported that expression of B3GALT5 is associated with cancer progression and can serve as cancer marker for diagnosis in gynecological cancers [10] and prognosis in hepatocellular carcinoma [11]. In breast cancer, tumor-associated glycans are usually associated with cell adhesion, migration, proliferation, tumor growth, and metastasis [12, 13]. In BCSCs, the expression of SSEA-3 has been reported and the B3GALT5-regulated SSEA-3 expression is crucial by its pro-survival activity [14, 15]. However, little is known regarding the clinical relevance of B3GALT5 in breast cancer subgroups and its biological function.

In this study, we aimed to delineate the clinical significance of B3GALT5 and to investigate its involvement in EMT and BCSCs of breast cancer.

## Material and methods

### Cell culture

Breast cancer cell line MDA-MB-231 was purchased from American Type Culture Collection (ATCC) and cultured in Dulbecco’s modified Eagle’s medium (DMEM) (Gibco Life Technologies, CA, USA) containing 100 units/ml penicillin and 100 μg/ml streptomycin sulfate, supplemented with 10% (v/v) fetal bovine serum (FBS) (growth medium) at 37 °C in 5% CO_2_. Monolayer cultures of H2K^d−^ ALDH^h^ cells sorted from BC0634 PDX tumor from a patient with luminal type (PR+) breast cancer were designated as AS-B634 as established in our previous study [16]. AS-B634 were cultured in minimum essential medium (MEM) (Gibco Life Technologies, CA, USA) containing 1 mM sodium pyruvate, 2 mM GlutaMAX, and 10 μg/ml insulin, supplemented with 10% (v/v) FBS at 37 °C in 5% CO_2_.

### Animal models

AS-B634 were transfected with scrambled negative control (Catalog#465372, Invitrogen) or *B3GALT5* siRNA (Catalog#314585B11, Invitrogen) for 72 h. Transfected cells were mixed with matrigel (356237, BD Bioscience, CA, USA) for subcutaneous injection into the mammary fat-pads of 6–8-week-old female NSG mice which were purchased from The Jackson Laboratory (Bar Harbor, ME, USA) and maintained under specific pathogen-free conditions at Laboratory Animal Center in Chang Gung University (Taoyuan, Taiwan). Tumor formation was monitored weekly after inoculation. The protocol for animal experimentation was approved by the Institutional Animal Care and Utilization Committee of Chang Gung Memorial Hospital at Linkou, Taoyuan, Taiwan (Permit number: CGU106-055).

### Patient samples

Human breast cancer tissues were obtained from the patients diagnosed between April 2005 and December 2012 in the department of surgery and pathology, at the Tri-Service General Hospital, Taipei, Taiwan. The use of human tissues for this study was approved by the Institutional Review Board of the Human Subjects Research Ethics Committee of Academia Sinica and Tri-Service General Hospital, Taipei, Taiwan. Written consent was obtained from patients before the collection of samples. Post-operation, human breast cancer tissues were collected for RNA extraction. The clinicopathological data of the patients were described in Supplementary Fig. S1. Tumor and adjacent non-tumor breast tissues from patients were obtained at the time of initial surgery and fully encoded to protect patient confidentiality.

### Small interfering RNA and transfection

Scrambled negative control small interfering RNA oligonucleotides (siRNA) and siRNA against B3GALT5 (HSS115774) and siRNAs against ZEB1 (HSS110548 and HSS186235) were purchased from ThermoFisher (CA, USA). The catalog number of si-Ctrl, si-B3GALT5-1, si-B3GALT5-2, si-ZEB1-1, and si-ZEB1-2 were #465372, #314585B11, #314585C01, #10620318, and #10620319, respectively. The siRNA transfection experiments were carried out using supermix reagent (ThermoFisher, CA, USA) with 100 nM siRNA according to the manufacturer’s instructions. After siRNA transfection for 48 h, cells were used for the indicated experiments.

### Plasmids and lentiviruses

For plasmid construction, full-length coding sequences of human B3GALT5 from B3GALT5 (NM_006057) Human Tagged ORF Clone (Origene, MD, USA) were cloned with pLenti6/V5 Directional TOPO Cloning Kit (ThermoFisher, CA, USA) according to the manufacturer’s instructions. Viral supernatant was collected 48 h after transfection and then filtered with 0.45-μm filters. MDA-MB-231 cells were transduced with lentiviruses carrying vector overexpressing B3GALT5 or control vector at a multiplicity of infection (MOI) of 10 in the presence of 8 μg/ml polybrene (Sigma-Aldrich, MO, USA) for 12 h. Cells were selected with 10 μg/ml blasticidin (Catalog #A1113903, ThermoFisher, CA, USA) for more than 1 month to generate stable clones of blasticidin-resistant cells.

### RNA preparation and quantitative reverse-transcription real-time PCR

Total RNA was extracted from cells with TRIzol reagent (Invitrogen, CA, USA) according to the manufacturer’s instructions. The cDNA was reverse-transcribed from 2 μg of RNA by using the Super-Script III First-Strand synthesis kit (Invitrogen, CA, USA). The 5 ng cDNA was applied to SYBR Green QPCR system. Glyceraldehyde-3-phosphate dehydrogenase (*GAPDH*) was used as endogenous controls. The SYBR probes for the detection of *B3GALT5* (Hs00707757_s1) and *GAPDH* (Hs99999905_m1) were purchased from Applied Biosystems. B3GALT5 (F) 5′-ATCAGGCAGCCATTCAGCAA-3′, (R) 5′-ACGTCGCCAGAAAACACGTA-3′; GAPDH (F) 5′-GTCTCCTCTGACTTCAACAGCG-3′, (R) 5′-ACCACCCTGTTGCTGTAGCCAA-3′; ZEB1 (F) 5′-GGCATACACCTACTC AACTACGG-3′, (R) 5′-TGGGCGGTGTAGAATCAGAGTC-3′. The Q-PCR assay was performed with Brilliant SYBR Green QPCR Master Mix (#04673484001, Roche, Basel, Switzerland) by 7500 Fast real-time PCR system (ThermoFisher, CA, USA). The relative amount of *GAPDH* mRNA was applied for normalization by using the comparative Ct method.

### Western blot

Western blot was performed according to regular protocol. Briefly, cell lysates of treated cells were extracted and transferred onto PVDF membranes. Membranes were blocked in PBS supplemented with 0.5% (v/v) Tween 20 (PBST) plus 10% (w/v) nonfat milk for 1 h followed by incubation with primary (1:100 to 2000) antibodies at 4 °C overnight. The membrane was then scanned by a Typhoon9400 Variable Mode Imager (GE Health Life Sciences, Uppsala, Sweden) to detect the fluorescent signals released from catalyzed ECF substrate (GE Healthcare, Little Chalfont, UK). Primary antibodies used were as follows: ZEB1 (Catalog #3396, Cell Signaling Technology), β-actin (Catalog #A5441, Sigma-Aldrich), β-catenin (Catalog #05-665, Sigma-Aldrich), histone (Catalog #MAB1276, Sigma-Aldrich), and GAPDH (Catalog #GTX100118, GeneTex). The results of Western blots were quantified by Image Quant 5.2 software (GE Healthcare, Little Chalfont, UK).

### Transwell cell migration and invasion assay

For migration assay, 4 × 10^4^ cells in 100 μl serum-free medium were seeded on top of the filter membrane (8.0 μm pores) in a transwell insert (6.5 mm diameter) (Corning, NY, USA). 10% FBS (v/v) was supplemented in medium and placed in the lower chamber as a chemoattractant. Cells were allowed to migrate at 37 °C for 15 h, and the transwell insert was removed from the plate for fixation and staining with crystal violet. The migrated cells on the underside of the filter membrane were viewed with an inverted microscope and counted for cell numbers. For invasion assay, similar procedures were performed but with 60 μl matrigel placed onto the upper chamber of transwell and incubated at 37 °C for 1 h before seeding the cells.

### Cell proliferation assay

At 48 h after siRNA transfection, cells were trypsinized and seeded at 1 × 10^5^ cells per well into 6-well plates. Cells were trypsinized for determination of viable cell number every 24 h (Countness™ II FL, Invitrogen, CA, USA).

### Mammosphere assay

Cultured cells were trypsinized and seeded at 2000 cells per well into 96-well low-attachment plates (Corning, NY, USA) and cultured in DMEM/F12 medium (Lonza, MD, USA) containing 0.4% (w/v) bovine serum albumin, 20 ng/ml basic fibroblast growth factor (PeproTech, NJ, USA), 20 ng/ml EGF (PeproTech), 5 μg/ml insulin, and B27 supplement (Gibco Life Technologies, CA, USA). The numbers of mammospheres (size > 50 μm diameter) were counted under an inverted microscope after 7 days’ culture.

### Statistical analysis

The prognostic performance of genes was calculated by the receiver operating characteristic curve. The Youden index (sensitivity + specificity − 1) was used to determine the optimal cut-off value for high versus low gene expression level. Survival curves were plotted by Kaplan–Meier method with the log-rank test applied for comparison. Quantitative data were expressed as mean ± SD or mean ± SEM as described in the figure legends. Statistical analysis was determined by Student’s *t* test, Mann–Whitney *U* test, or Kruskal–Wallis test with Dunn multiple comparison for significance. * = *P* < 0.05, ** = *P* < 0.01, *** = *P* < 0.001.

## Results

### High expression of B3GALT5 in adjacent non-tumor tissue is an independent poor prognostic factor for breast cancer

**Fig. 1:**
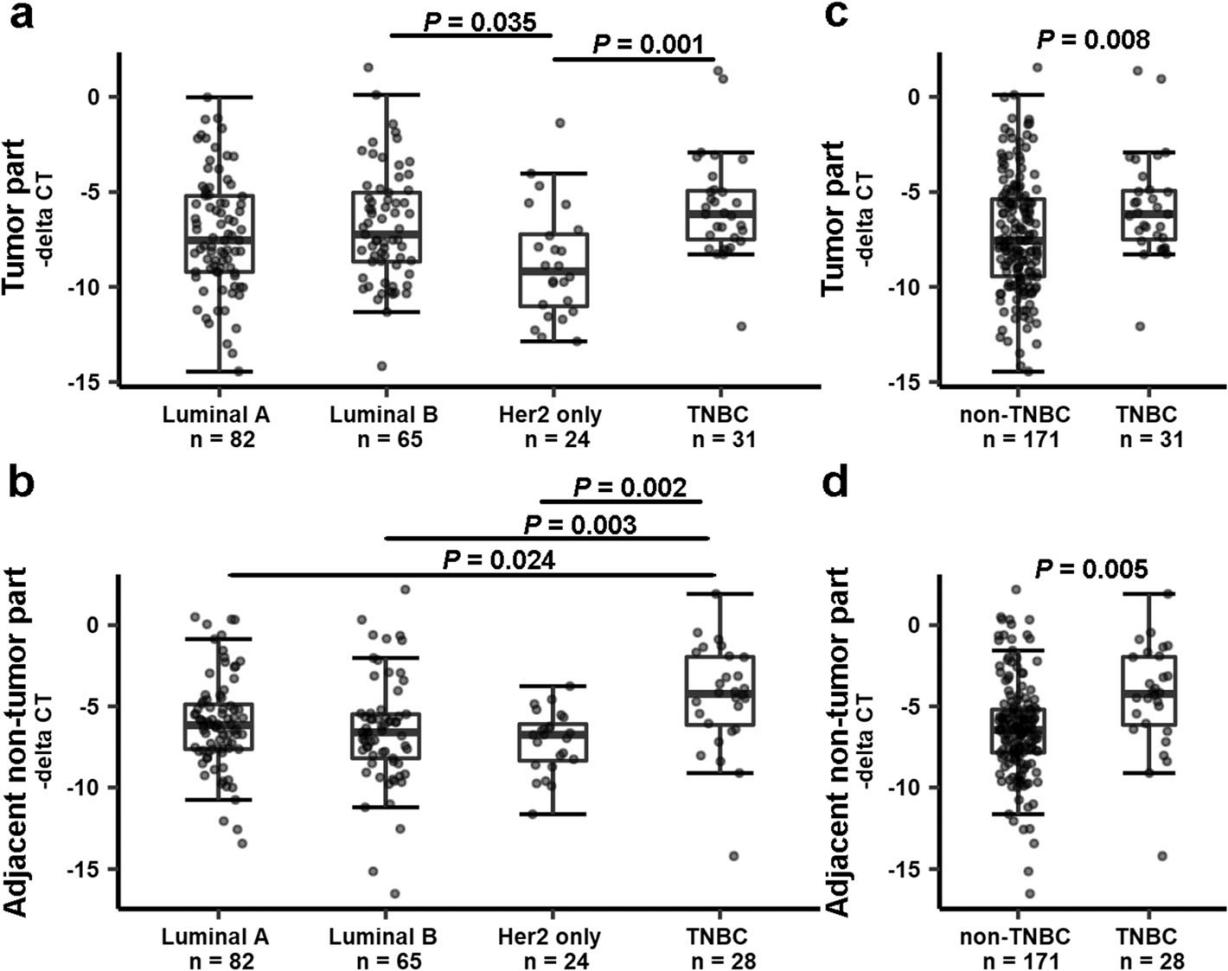
Expression levels of *B3GALT5* in tumor and adjacent non-tumor parts of breast cancer tissues. The mRNA expression levels of *B3GATL5* in breast cancer tissues (*n* = 202) were determined by qRT-PCR. The CT value of each specimen was normalized to the average of control and presented as minus delta threshold cycle (−dCt). **a**, **b** The values of −deltaCt of *B3GALT5* in tumor part (**a**) and adjacent non-tumor part (**b**) of cancer tissues of breast cancer patients with luminal A, luminal B, Her2, and TNBC types were compared by Kruskal–Wallis test with Dunn multiple comparison. **c**, **d** The values of −deltaCt of *B3GALT5* in tumor part (**a**) and adjacent non-tumor part (**b**) of cancer tissues of breast cancer patients with luminal A, luminal B, Her2, and TNBC types were compared by Kruskal–Wallis test with Dunn multiple comparison. **c**, **d** The values of −deltaCt of *B3GALT5* in tumor part (**c**) and adjacent non-tumor part (**d**) of cancer tissues of breast cancer patients with non-TNBC and TNBC types were compared by Mann–Whitney *U* test and *t* test (not equal variance), respectively

To explore the clinical relevance of B3GALT5 expression in breast cancers, we performed qRT-PCR analysis to examine *B3GALT5* mRNA expression levels in tumor and adjacent non-tumor tissues obtained from 202 breast cancer patients. Their clinical characteristics and demographic information were summarized in Supplementary Fig. S1. The *B3GALT5* mRNA levels in tumor (Fig. 1a and c) and adjacent non-tumor tissue (Fig. 1b and d) were shown according to different molecular subtypes of breast cancer. In the tumor tissue, *B3GALT5* mRNA levels were significantly higher in the luminal B subtype (*P* = 0.035) and TNBC subtype (*P* = 0.001) than the HER-2-positive subtype (Fig. 1a). Similarly, significantly higher *B3GALT5* mRNA levels were found in TNBC subtype (*P* = 0.008) than non-TNBC subtype (Fig. 1c). As to the adjacent non-tumor tissue, we found more significantly higher *B3GALT5* mRNA levels in TNBC subtype than the other three subtypes (*P* = 0.002 vs HER-2-positive subtype; *P* = 0.024 vs luminal A subtype; *P* = 0.003 vs luminal B subtype) (Fig. 1b). Consistently, the *B3GALT5* mRNA levels were significantly higher in TNBC subtype (*P* = 0.005) than non-TNBC subtype (Fig. 1d). On the other hand, the *B3GALT5* mRNA levels in tumor and adjacent non-tumor tissue showed no significant correlation with either stages or grades of breast cancer (Supplementary Fig. S2).

We further conducted univariate (Table 1) and multivariate (Table 2) Cox proportional hazard regression analyses to assess the impact of the expression levels of *B3GALT5* on breast cancer recurrence and patient survival. The results indicate that relapse-free survival (RFS) correlated with age greater than 45 years (HR = 2.90, *P* = 0.02), and the higher expression of *B3GALT5* in adjacent non-tumor parts (HR = 5.08, *P* = 1.7E−07) and tumor part (HR = 2.54, *P* = 3.8E−03). The overall survival (OS) correlated with the higher AJCC cancer stages III–IV (HR = 2.81, *P* = 2.5E−04), and higher expression of *B3GALT5* in adjacent non-tumor part (HR = 2.63, *P* = 4.7E−03). Similarly, in multivariate analysis, RFS and OS were both correlated with the higher AJCC cancer stages III–IV (HR = 2.40, *P* = 7.9E−03, and HR = 3.06, *P* = 1.0E−04, respectively) and expression of *B3GALT5* in adjacent non-tumor tissue (HR = 4.20, *P =* 5.1E−05, and HR = 2.50, *P* = 0.02, respectively).

**Table 1:** Univariate Cox proportional regression analysis of factors associated with breast cancer recurrence and survival

Variable	RFS		OS	
	HR	*P* value	HR	*P* value
**Age**				
≥ 45	2.90	**0.02**	2.06	0.06
**Grade**				
II	1.9E+7	1.00	2.34	0.41
III	3.2E+7	1.00	4.10	0.16
**AJCC**				
III–IV	1.74	0.08	2.81	**2.5E−04**
**Subtype**				
Luminal A	1.14	0.80	1.14	0.77
Luminal B	1.23	0.69	1.04	0.94
TNBC	2.01	0.20	1.24	0.69
**TNBC**				
TNBC	1.75	0.10	1.15	0.72
**B3GALT5_N**				
High	5.08	**1.7E−07**	2.63	**4.7E−03**
**B3GALT5_T**				
High	2.54	**3.8E−03**	1.50	0.26

*B3GALT5_N* adjacent non-tumor part, *B3GALT5_T* tumor part

**Table 2:** Multivariate Cox proportional regression analysis of factors associated with breast cancer recurrence and survival

Variable	RFS		OS	
	HR	*P* value	HR	*P* value
Age ≥ 45	2.37	0.08	1.84	0.14
Subtype Luminal A	0.98	0.97	1.29	0.59
Subtype Luminal B	1.02	0.97	1.07	0.90
Subtype TNBC	1.16	0.80	1.05	0.93
AJCC III–IV	2.40	**7.9E−03**	3.06	**1.0E−04**
B3GALT5_N High	4.20	**5.1E−05**	2.50	**0.02**
B3GALT5_T High	1.80	0.10	0.91	0.82

*B3GALT5_N* adjacent non-tumor part, *B3GALT5_T* tumor part

**Fig. 2:**
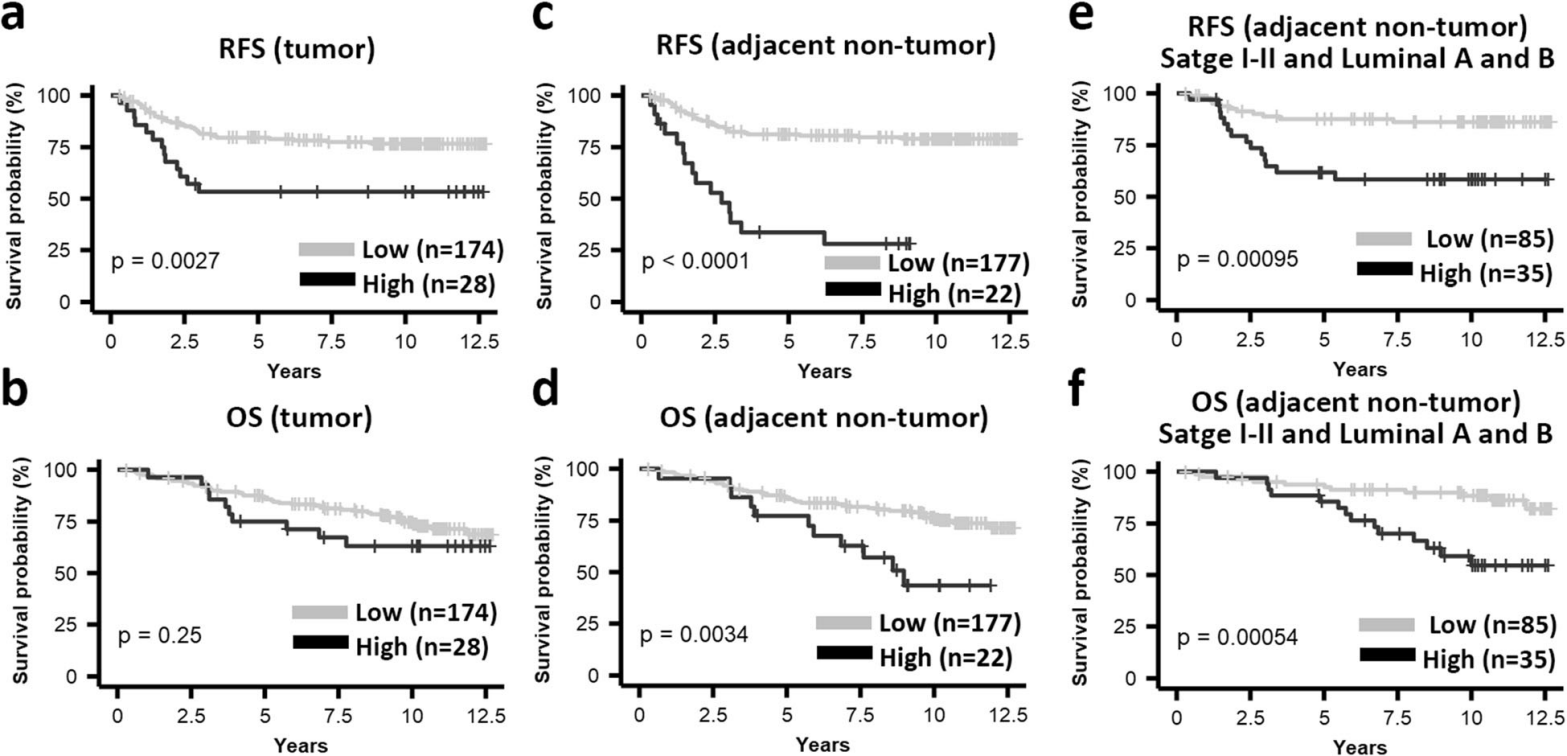
High expression of *B3GALT5* in breast cancer tissue correlates with poor clinical outcome. Relapse-free survival (RFS) and overall survival (OS) comparing the 202 breast cancer patients with high (black) and low (gray) expression of *B3GALT5* in tumor and adjacent non-tumor parts was analyzed. **a**–**d** Kaplan–Meier plots of RFS (**a**, **c**) and OS (**b**, **d**) for patients with breast cancer in relation to the expression levels of *B3GALT5* in tumor parts (**a**, **b**) and adjacent non-tumor parts (**c**, **d**) of the cancer tissues. **e**, **f** Kaplan–Meier plots of RFS (**e**) and OS (**f**) for patients with stages I–II and luminal A and B breast cancer in relation to the expression levels of *B3GALT5* in adjacent non-tumor parts of the cancer tissues. *P* values were calculated by log-rank test

Therefore, to investigate whether high expression of *B3GALT5* was a significant predictor of recurrence of breast cancer, we performed Kaplan–Meier analysis on 202 breast cancer patients. The RFS in patients with high expression of *B3GALT5* in tumor tissue was significantly worse than those with low expression of *B3GALT5* (*P* = 0.002) (Fig. 2a), while similar trend was observed for OS (Fig. 2b) although it did not reach statistical significance (*P* = 0.25). The adverse impact of high expression of *B3GALT5* found on RFS is consistent with Kaplan–Meier analysis using GSE1456 database, which reveals that breast cancer patients with higher *B3GALT5* expression has shorter RFS than those with low *B3GALT5* expression (*P* = 0.007) (Supplementary Fig. S3). To our surprise, Kaplan–Meier plots of adjacent non-tumor tissue also show that both RFS (Fig. 2c) and OS (Fig. 2d) in patients with high expression of *B3GALT5* were significantly worse than those with low expression (RFS: *P* < 0.0001; OS: *P* = 0.003). We further determined the prognostic significance of *B3GALT5* expression in both tumor and adjacent non-tumor tissue among different subsets of breast cancer patients, including stages I–II (*n* = 158), stages I–II and luminal A (*n* = 68), and stages I–II and luminal B (*n* = 52), as shown in the Kaplan–Meier analysis of RFS (Supplementary Fig. S4) and OS (Supplementary Fig. S5). The adverse impacts of high expression of *B3GALT5* in non-tumor parts on RFS (Fig. 2e) and OS (Fig. 2f) were found most significant in patients with early stage and luminal A and B subtypes (*P* = 9.5E−04 and *P* = 5.4E−04, for RFS and OS, respectively). In addition, in tumor tissue of TNBC patients (Supplementary Fig. S6), high expression of *B3GALT5* had adverse impact on RFS (*P* = 0.004) but not OS. Taken together, the results indicated the adverse prognostic significance of high expression of *B3GALT5* in breast cancer, especially for patients with early stage hormonal receptor positive tumors.

### Knockdown of B3GALT5 attenuates cell migration and invasion in breast cancer stem cells derived from BC0634 PDX

**Fig. 3:**
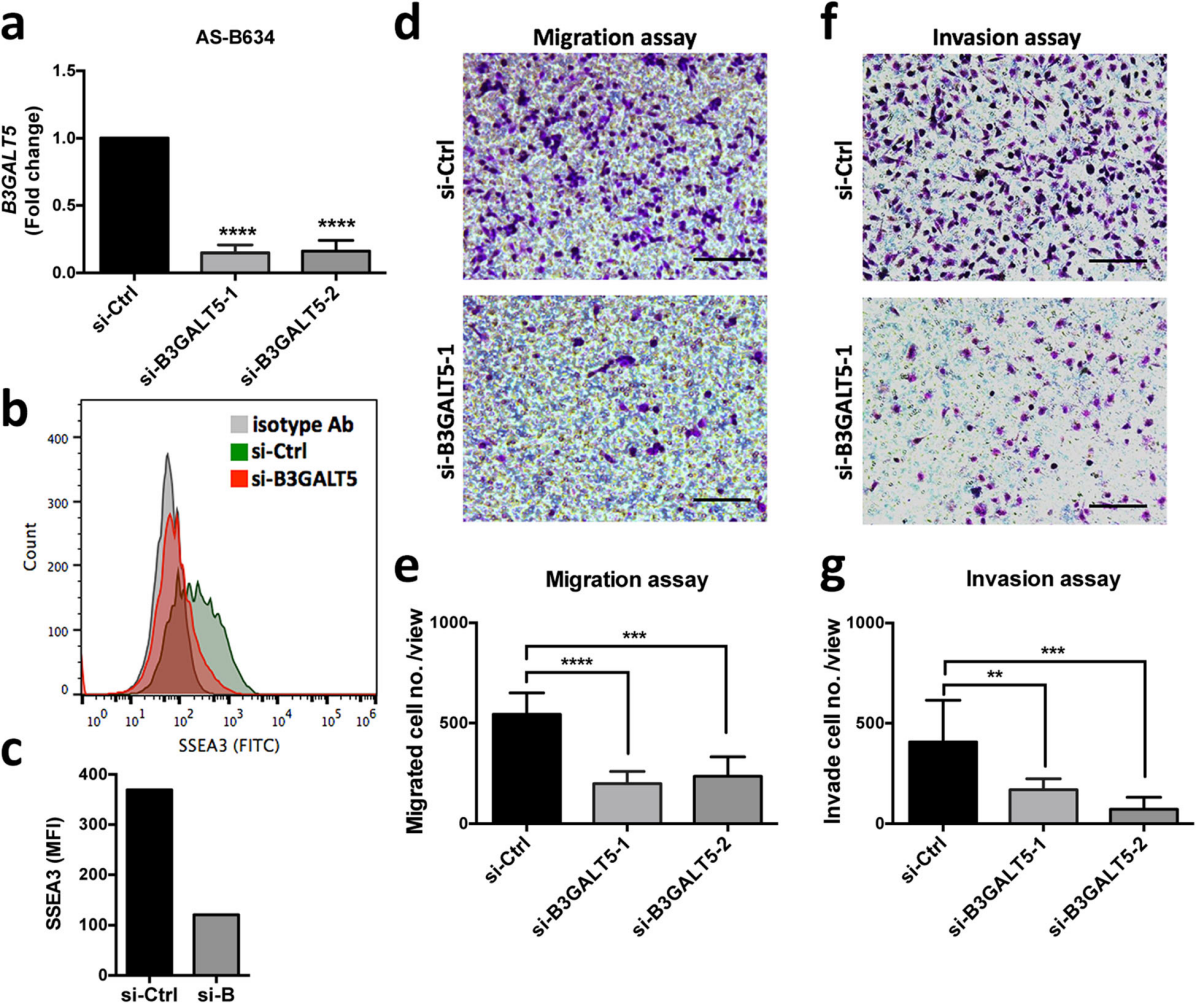
Effects of *B3GALT5* knockdown on cell migration and invasion. **a** mRNA expression of *B3GALT5* in AS-B634 cells transfected with control siRNA (si-Ctrl) or B3GALT5 siRNA (si-B3GALT5-1 and si-B3GALT5-2) as determined by qRT-PCR. ****P < 0.0001 compared to the si-Ctrl control by *t* test. **b**, **c** Expression of B3GALT5 downstream target SSEA-3 detected by flow cytometry. **d**, **f** Representative pictures of transwell cell migration and invasion assays of AS-B634. Scale bar 100 μm. **e**, **g** Quantitation of migrated or invaded cells per well. Data were shown as mean ± SD for triplicates. **** *P *< 0.0001, ***P < 0.001, **P < 0.01 by *t* test

To elucidate the biological functions of B3GALT5 in breast cancer that contribute to its adverse prognostication, we first evaluated the impacts of B3GALT5 knockdown on migration and invasion of breast cancer cells. We used AS-B634 which were cultured from BCSCs harvested from BC0634 patient-derived xenograft (PDX) tumor from a patient with luminal type (PR+) breast cancer established in our laboratory for silencing B3GALT5 and breast cancer cell line MDA-MB-231 for overexpressing B3GALT5 to investigate its roles in breast cancer. Successful silencing of B3GALT5 with two clones of siRNA in AS-B634 cells was confirmed by reduction of *B3GALT5* mRNA compared with si-Control (*P* < 0.001) (Fig. 3a) and downregulation of its downstream target SSEA-3, as detected by flow cytometry (Fig. 3b and c). As shown in Figs. 3d–g, transwell cell migration (Fig. 3d and e) and invasion (Fig. 3f and g) assays revealed that silencing of *B3GALT5* significantly reduced migration and invasion ability of AS-B634 compared to si-Control.

### Overexpression of B3GALT5 promotes cell migration and invasion in MDA-MB-231 breast cancer cells

**Fig. 4:**
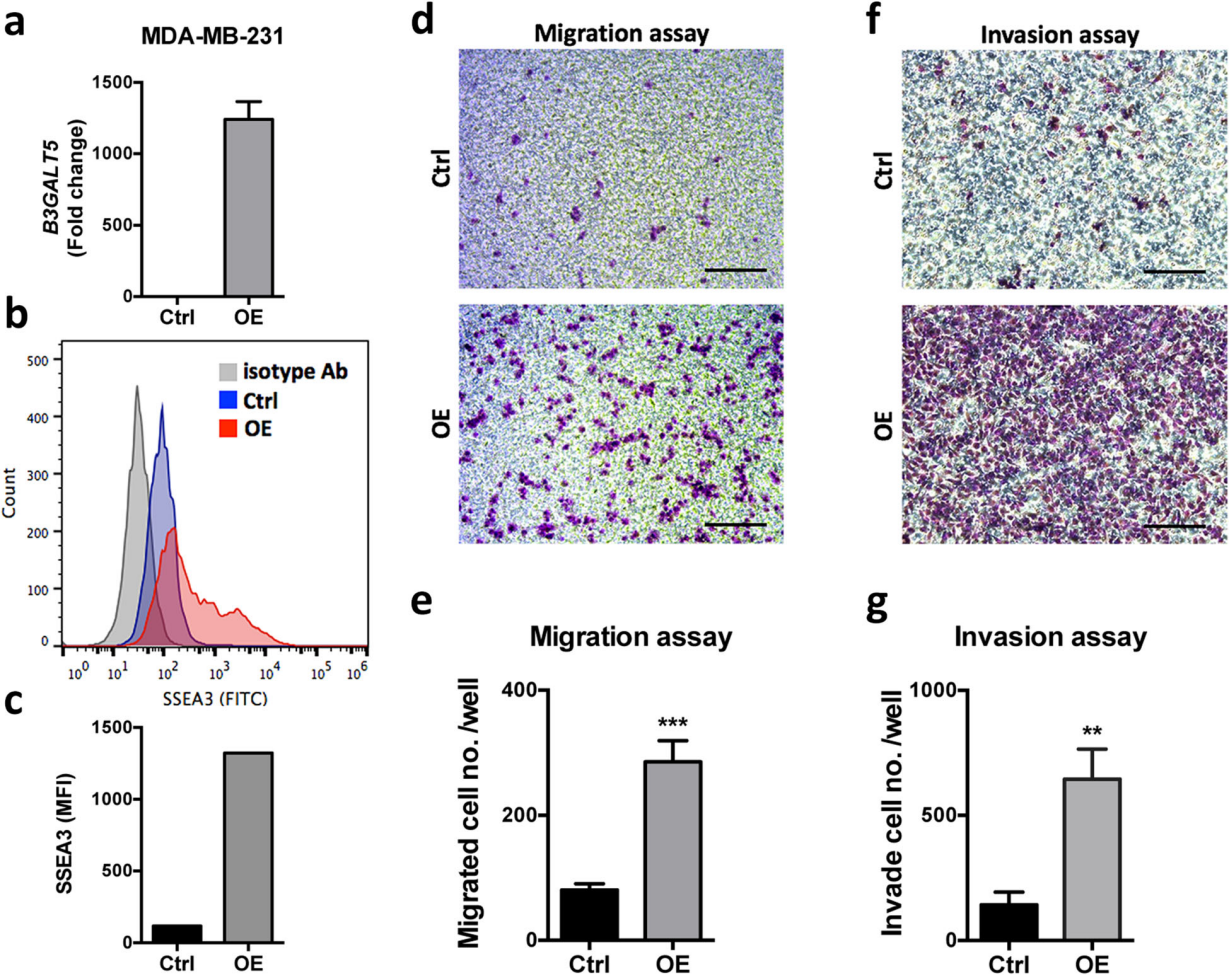
Effects of *B3GALT5* overexpression on cell migration and invasion. **a** mRNA expression of *B3GALT5* in MDA-MB-231 cells transfected with vector control plasmids (Ctrl) or B3GALT5 overexpression plasmids (OE) as determined by qRT-PCR. **b**, **c** Expression of B3GALT5 downstream target SSEA-3 detected by flow cytometry. **d**, **f** Representative pictures of transwell cell migration and invasion assays of MDA-MB-231. Scale bar 100 μm. **e**, **g** Quantitation of migrated or invaded cells per well. Data were shown as mean ± SD for three independent experiments. ***P < 0.001, **P < 0.01 by *t* test

We then examined the effects of overexpression of B3GALT5 on the migration of breast cancer cells. Stable clone of MDA-MB-231 overexpressing B3GALT5 by infection with lentiviruses carrying vector expressing B3GALT5. Efficiency of B3GALT5 overexpression was confirmed by significant increase in mRNA levels (*P* < 0.001) (Fig. 4a) and upregulation of SSEA-3 expression by flow cytometry (Fig. 4b and c). As shown in Figs. 4d–g, overexpression of B3GAL5 in MDA-MB-231 cells significantly potentiates the migration (Fig. 4d and e) (*P* < 0.001) and invasion (Fig. 4f and g) (*P* < 0.01) activity. Taken together, these findings suggest that B3GALT5 plays a key role in the migration and invasion ability in breast cancer.

### B3GALT5 regulates epithelial-to-mesenchymal transition (EMT), ZEB1 expression, and β-catenin nuclear translocation

**Fig. 5:**
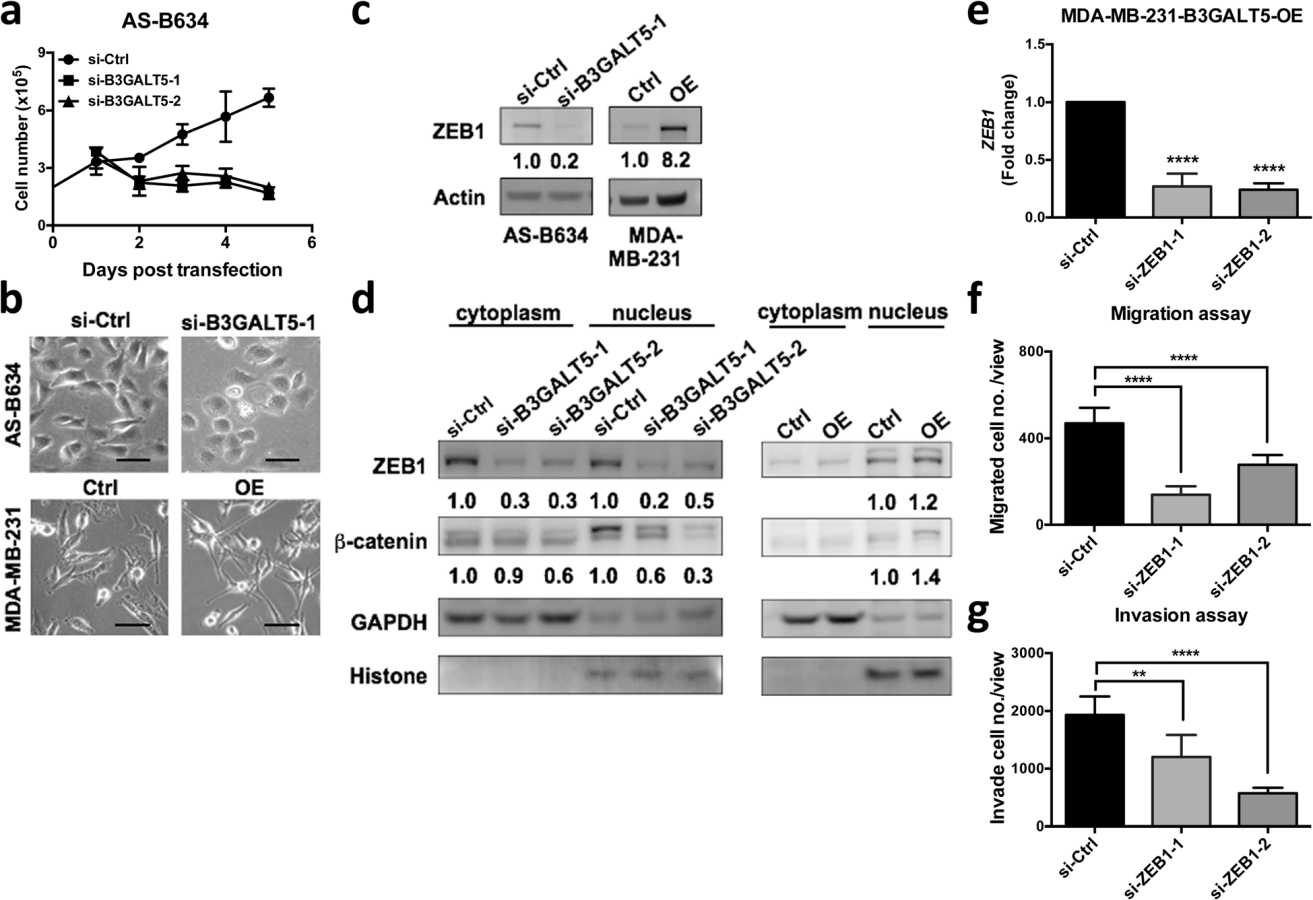
Knockdown or overexpression of *B3GALT5* altered cell proliferation, morphology and expression of ZEB1 and β-catenin. **a** AS-B634 cells were transfected with control siRNA (si-Ctrl) or B3GALT5 siRNA (si-B3GALT-1 and si-B3GALT-2) and cultured for 5 days. Cell numbers were counted every day. **b** Phase contrast photographs of AS-B634 cells transfected with control siRNA (si-Ctrl) or B3GALT5 siRNA (si-B3GALT5-1) and MDA-MB-231 cells transfected with vector control plasmids (Ctrl) or B3GALT5 overexpression plasmids (OE). Scale bar 50 μm. **c** ZEB1 expression in AS-B634 and MDA-MB-231 as determined by western blotting. The expression of ZEB1 was normalized to actin and presented as relative fold change. **d** Cytoplasmic and nuclear ZEB1 and active β-catenin expression in AS-B634 and MDA-MB-231 as determined by western blotting. The expression of cytoplasmic or nuclear ZEB1 and active β-catenin was normalized to GAPDH or Histone 1, respectively, and presented as relative fold change. **e**–**g** MDA-MB-231 B3GALT5 overexpressing cells (OE) were transfected with control siRNA (si-Ctrl), si-ZEB1-1 or si-ZEB1-2 (**e**). Migration (**f**) and invasion (**g**) assays were performed. **f**, **g** Data were shown as mean ± SD. ****P < 0.0001, **P < 0.01 by *t* test. Three independent experiments were performed in each study

Along with the above-mentioned migration and invasion studies, we found knockdown of B3GALT5 also significantly attenuated the growth of AS-B634 cells (Fig. 5a) indicating that B3GALT5 might contribute to cell proliferation. We also noticed that silencing of B3GALT5 expression in AS-B634 cells substantially altered the cell morphology from fibroblast-like morphology with triangular shaped cells and tapering end to epithelial like round or cuboidal cells with loss of tapering ends (Fig. 5b). In contrast, overexpression of B3GALT5 in MDA-MB-231 cells altered the cell morphology into longer and narrower fibroblastic morphology (Fig. 5b). These morphological changes suggest that B3GALT5 might be involved in the epithelial-to-mesenchymal transition (EMT) process. To further determine if B3GATL5 regulates EMT, we examined the expression of zinc finger E-box binding homeobox transcription factor 1 (ZEB1), which is the key factor to activate EMT and promote metastasis in cancers [17, 18]. As shown in Fig. 5c, downregulation of B3GALT5 in AS-B634 cells decreased ZEB1 expression to 0.2 fold of si-Control whereas overexpression of B3GAL5 in MDA-MB-231 cells increase ZEB1 expression to 8.2 fold of vector control. We further investigated the possible mechanism by which the expression of ZEB1 is regulated. It has been shown that activated β-catenin could undergo nuclear translocation and induce ZEB1 transcription [19]. In our study, decreased ZEB1 accompany with reduced active form of β-catenin were found upon B3GALT5 knockdown. Overexpression of B3GALT5 in MDA-MB-231 cells showed the opposite results. We further found the subcellular localization of ZEB1 upstream regulator β-catenin was regulated by B3GALT5. B3GALT5 silencing reduced the nuclear translocation of active β-catenin (Fig. 5d, left panel), whereas B3GALT5 overexpression increased it (Fig. 5d, right panel). These observations suggest that B3GALT5 might regulate ZEB1 via β-catenin and thereby modulating ZEB1-induced EMT. We further examined the effect of knockdown of ZEB1 in migration and invasion abilities of B3GALT5 overexpressing cell. As shown in Fig. 5e, ZEB1 was successfully downregulated by either siRNA-1 or siRNA-2. Silencing of ZEB1 in B3GALT5 overexpressing MDA-MB-231 cells by two ZEB1 siRNAs significantly reduced their abilities in migration (Fig. 5f) and invasion (Fig. 5g). These findings indicate that B3GALT5 modulate migration and invasion via ZEB1.

### B3GALT5 contributes to mammosphere formation in vitro and tumor growth and metastasis in vivo

**Fig. 6:**
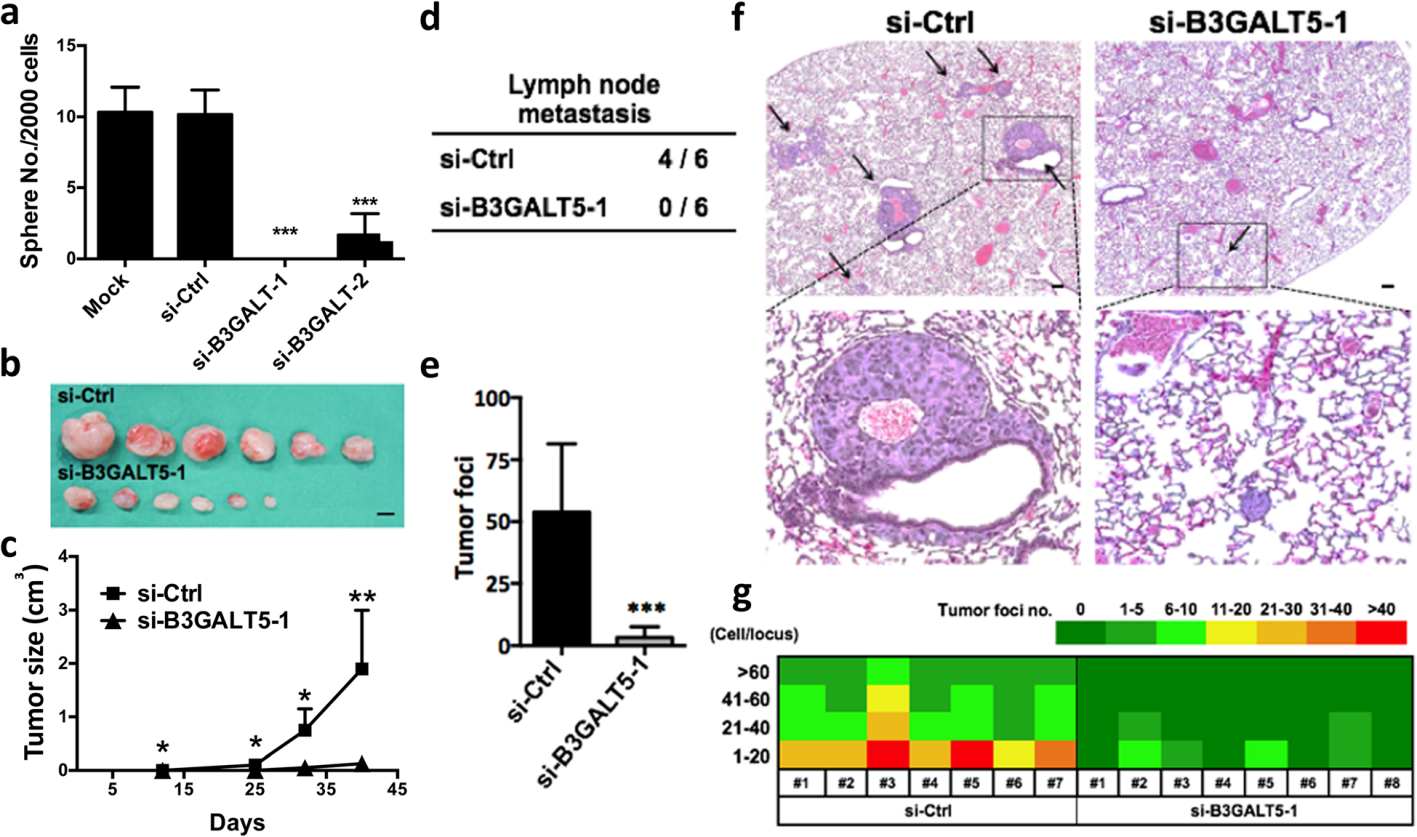
Knockdown of *B3GALT5* inhibits mammosphere formation, tumor growth, and metastasis of AS-B634 breast cancer cells. **a** AS-B634 cells transfected with control siRNA (si-Ctrl) or B3GALT5 siRNA (si-B3GALT5-1 and si-B3GALT5-2) and the numbers of mammosphere per 2000 cells were graphed as histogram. Data were shown as mean ± SD for three independent experiments. ***P < 0.001 compared to the si-Ctrl control by *t* test. **b** Photographs of tumors excised 6 weeks after mammary fat pad injection of AS-B634 cells transfected with control siRNA (si-Ctrl) or B3GALT5 siRNA (si-B3GALT5-1) in NSG mice (*n* = 6 for each group). Scale bar 1 cm. **c** Tumor growth as measured at the indicated days. *P < 0.05, **P < 0.01 by *t* test. **d**, **e** The frequency of lymph node metastasis (**d**) and the colony number (**e**) of lung metastasis detected at the time of sacrifice at 6 weeks. ***P < 0.001 by *t* test. **f** Spontaneous lung metastasis at 6 weeks after injection of AS-B634 cells, as shown by histology (upper panel) and enumeration of tumor foci (lower panel). Metastatic lesions were indicated by arrows. Inserts show × 20 magnifications. Scale bar 100 μm. **g** Distribution of number and size of metastatic foci in individual mouse shown as heatmap. Three independent experiments were performed in each study

In suspension cultures, BCSCs could be enriched as mammospheres [20] which is an indicator for functional characterization of cancer stem cell with strongly tumor initiating potential [21]. To understand the possible involvement of B3GALT5 in maintaining BCSC-like property, mammosphere formation capacity was analyzed in *B3GALT5* knockdown cell lines. Knockdown of B3GALT5 in AS-B634 cells resulted in a significant reduction in the number of spheres compared to si-Control, *P* < 0.001 (Fig. 6a and Supplementary Fig. S7). These results thus suggest that B3GALT5 is engaged in enhancing mammosphere formation.

To further investigate whether B3GALT5 affects tumor growth in vivo, breast cancer with B3GALT5-silenced AS-B634 cells were injected into mammary fad of NSG mice. As shown in Fig. 6b and c, knockdown of B3GALT5 significantly suppressed tumor growth rate and decreased tumor size compared to si-Control. We also noted the absence of lymph node metastasis in mice bearing tumor of *B3GALT5*-knockdown AS-B634 cells, as compared to lymph node metastasis in 4/6 mice bearing control AS-B634 tumor (Fig. 6d). Consistently, the number of metastatic lesions in the lungs in the mice bearing tumor of *B3GALT5*-knockdown AS-B634 cells is significantly decreased compared with si-Control, *P* < 0.001 (Fig. 6e and f). The lung metastatic foci were further enumerated and grouped by their sizes as determined by their cell numbers per locus (Supplementary Fig. S8). As shown in the heatmap (Fig. 6g), knockdown of *B3GALT5* results in an overall reduction in the number and size of metastatic foci. These results suggested that B3GALT5 contributes to tumor growth and metastasis in vivo.

## Discussion

In the present study, we provide the first evidence for the predictive value of *B3GALT5* mRNA expression in the outcome of breast cancer patients. Higher expression of *B3GATL5* in adjacent non-tumor part of the breast cancer tissue from patients with early stage and luminal type strongly correlates to worse RFS and OS. This finding suggests that *B3GATL5* expression may be a useful biomarker for the decision-making of treatment options, e.g., aggressive treatments may be considered for those who have high B3GATL5 expression. To date, there is only one report showing B3GALT5 expression in breast carcinoma tissues as determined by immunohistochemistry (IHC) [22] positively correlated with the stages and poor disease-free survival. However, there was no information on the prognostic value of B3GALT5 expression in early-stage disease nor the difference of its expression among molecular subtypes. Although IHC is widely used and less expensive as compared to genomics-based technologies, the available antibodies for B3GALT5 including the antibody used in this study displayed cross reactivities with many undefined proteins and thus may not accurately reflect B3GALT5 expression levels.

Here we showed significant higher expression of *B3GALT5* in TNBC subtype of breast cancer tissues and provided the first evidence for the poor prognostic significance of *B3GALT5* expression in early stage breast cancer. To date, only 2 assays have been clinically validated and approved by US Food and Drug Administration (FDA) for guiding the treatment of early-stage breast cancer (ESBC): the 70-gene signature (MammaPrint®) and the 21-gene Recurrence Score (RS) assay (Oncotype DX®) [23, 24]. MammaPrint® is the first assay to be approved by US FDA to predict the clinical outcome of women with ER-positive or negative, lymph node-negative breast cancer [25]. Oncotype DX® is a diagnostic assay that quantifies the likelihood of distant breast cancer recurrence in women with newly diagnosed ESBC. Oncotype DX® is recommended as the treatment guidelines by the American Society of Clinical Oncology (ASCO) [26]. In patients with ER-positive ESBC, the benefit of adjuvant chemotherapy is often questionable and may not apply to all patients. With the help of Oncotype DX®/ MammaPrint®, the treatment decision-making might be refined to personalized plan and spare patients from the unnecessary treatment-related adverse events. However, both assays are quite complex and costly. Once the prognostic impact of B3GALT5 in early-stage breast cancer could be validated in further large clinical cohort, B3GALT5 might provide a novel simple and feasible candidate gene for predication of recurrent risk of ESBC.

Mechanistically, we found B3GALT5 could regulate cell proliferation, migration, and invasion through EMT activator ZEB1 in breast cancer cells as well as other properties of BCSCs, such as mammosphere formation, tumor initiation, and metastasis. ZEB1 is implicated as a prime element of a network of transcription factors that control cell proliferation, EMT, and cancer metastasis [27, 28] in various cancers including breast cancer [29, 30], pancreatic cancer [17], and lung cancer [31]. We found that overexpression of B3GALT5 altered cell morphology toward EMT and increased ZEB1 expression, whereas knockdown of B3GALT5 reduced EMT and cell proliferation as well as decreased ZEB1 expression. Given that Wnt/β-catenin signaling regulated EMT and might act as upstream of ZEB1 [19], we further demonstrated that B3GATL5 overexpression induced the nuclear translocation of active β-catenin along with ZEB1 upregulation and vice versa upon knockdown of B3GALT5.

Previous report of Cheung et al. [15] showed knockdown of B3GALT5 induces apoptosis in breast cancer cells. Chuang et al. [22] further demonstrated knockdown of B3GALT5 in breast cancer cells disrupts caveolin-1 and focal adhesion kinase complex to induce apoptosis via dissociation of RIP from the complex to induce Fas-dependent pathways. We herein provide further experimental evidence that B3GATL5 regulates EMT and cell proliferation in not only breast cancer cells but also BCSCs via activation of β-catenin and EMT regulator ZEB1. This is, to our knowledge, the first report linking B3GALT5 to EMT and β-catenin/ZEB1 axis in breast cancer. Moreover, we provided the first evidence for the important role of B3GALT5 in lymph node and lung metastasis of breast cancer, using PDX-derived BCSCs implantation model. Knockdown of B3GALT5 in BCSCs AS-B634 significantly retarded tumor growth and reduced metastasis. The results are in agreement with a recent report showing that ZEB1-driven EMT program may contribute to early nodal metastasis in breast cancer patients [32, 33]. The molecular regulation of B3GALT5 on β-catenin activation and ZEB1 expression is also currently under extensive investigation.

The molecular targets of B3GALT5 that contribute to cell proliferation, EMT, and metastasis have not yet been elucidated. B3GALT5 was reported to participate in the synthesis of histo-blood group antigens Lewis a (Le(a)), Lewis b (Le(b)), and sialyl-Lewis a (sLe(a)) [34]. Of note, sLe(a) is not only the selectin ligand involved in cancer metastasis but also the epitope of tumor marker CA 19.9 antigen which is presumed to be synthesized by B3GALT5 [35, 36]. Greater expression of sLe(a) or CA 19-9 correlates with metastasis and poor outcome in colon cancer patients [37]. Whether B3GALT5 directly regulates the activation of β-catenin/ZEB1 axis via glycosylation or indirectly via other target glycoprotein/glycolipid is of worth for further study.

Notably, we found the expression of *B3GALT5* in adjacent non-tumor part of the breast cancer tissue to be more significantly correlated to clinical outcome when compared to the tumor part. This is in line with studies which also showed that expression of ZEB1- and ZEB1-associated genes were significantly higher in adjacent normal tissue than in primary tumor [32, 33]. A similar report demonstrated the gene expression in histologically normal-appearing tissue adjacent to prostate tumor was of significance for the prediction of clinical recurrence [38]. The studies suggested that there is a biological field effect in primary cancer that might be a marker for aggressive disease. The “field cancerization” theory was first described in 1953 by Slaughter et al. and has long been debated [39]. A recent comprehensive study analyzed the transcriptomes of healthy, normal tissue adjacent to the tumor, and tumor tissues in 6506 samples across eight tissues and corresponding tumor types and found normal tissue adjacent to the tumor transcriptomically represented a unique intermediate state between healthy and tumor. Moreover, they demonstrated a pan-cancer mechanism of pro-inflammatory signals from the tumor stimulates an inflammatory response in the adjacent endothelium [40]. Thus, it is possible that the clinical significance of high *B3GALT5* expression in adjacent non-tumor part of the cancer tissues might be associated with higher ZEB1 expression and other important but unrevealed role of B3GALT5 in regulation of tumor microenvironment to facilitate tumor progression. Further in-depth study will be needed to clarify the molecular mechanisms involved.

## Conclusions

We have demonstrated that high *B3GALT5* expression serves as a poor prognostic marker for breast cancer patients including those with early stage diseases. Our studies have provided novel finding that *B3GALT5* plays a role in facilitating lymph node and lung metastasis through β-catenin/ZEB1 pathway, suggesting B3GATL5 might be potential target for tackling BCSCs, cell proliferation, and EMT in breast cancer.

## Supplementary Information

**Additional file 1: Figure S1.** Clinical and pathological characteristics of 202 breast cancer patients. **Figure S2.** Correlation between *B3GATL5* expression levels and stage or grade of breast cancers. Tumor (a and b) or adjacent non-tumor (c and d) tissues from patients with grade I, II, and III (a and c) or stage I, II, and III-IV (b and d) were analyzed for expression of *B3GALT5* mRNA by using qRT-PCR. Statistical analysis was performed One-way ANOVA analysis. **Figure S3.** Expression levels of *B3GALT5* in tumor of breast cancer tissues. The GSE1456 dataset was used to plot the survival curve comparing the patient with high (black) and low (gray) expression of *B3GALT5*. **Figure S4.** Higher expression of *B3GALT5* in breast cancer tissue correlates with poor clinical outcome (RFS). Kaplan-Meier plots of relapse-free survival (RFS) comparing the 202 breast cancer patients with high (black) and low (gray) expression of *B3GALT5* in tumor (a-c) and adjacent non-tumor parts (d-f) of patients with stage I-II (a and d), and stage I-II & Luminal A (b and e), and stage I-II & Luminal B (c and f) breast cancer were analyzed. **Figure S5.** Higher expression of *B3GALT5* in breast cancer tissue correlates with poor clinical outcome (OS). Kaplan-Meier plots of overall survival (OS) comparing the 202 breast cancer patients with high (black) and low (gray) expression of *B3GALT5* in tumor (a-c) and adjacent non-tumor parts (d-f) of patients with stage I-II (a and d), and stage I-II & Luminal A (b and e), and stage I-II & Luminal B (c and f) breast cancer were analyzed. **Figure S6.** Kaplan-Meier analyses of TNBC patients. Kaplan-Meier plots of RFS (a and c) or OS (b and d) comparing the TNBC breast cancer patients with high (black) and low (gray) expression of *B3GALT5* in tumor (a and b) and adjacent non-tumor parts (c and d) were analyzed. **Figure S7.** Knockdown of *B3GALT5* inhibits mammosphere formation. AS-B634 cells transfected with control siRNA (si-Ctrl) or B3GALT5 siRNA (si-B3GALT5-1 and -2) and the representative pictures of mammosphere were shown. Scale bar: 250 μm. **Figure S8.** Quantification of lung metastasis. Lung sections were stained with H&E. Counting of the pre-metastatic colonies were based on the cell numbers per colony. Representative pictures were shown. Scale bar: 100 μm.

## Data Availability

All data generated or analyzed during this study are included in this published article and its supplementary information files.

## Abbreviations

B3GALT5: β1,3-Galactosyltransferase 5
BCSCs: Breast cancer stem cells
EMT: Epithelia-to-mesenchymal transition
OS: Overall survival
PDX: Patient-derived xenograft
RFS: Relapse-free survival
SSEA-3: Stage-specific embryonic antigen-3
ZEB1: Zinc finger E-box binding homeobox transcription factor 1

## References

[1] Ferlay J, Soerjomataram I, Dikshit R, Eser S, Mathers C, Rebelo M, Parkin DM, Forman D, Bray F (2015). Cancer incidence and mortality worldwide: sources, methods and major patterns in GLOBOCAN 2012. Int J Cancer.

[2] Harbeck N, Penault-Llorca F, Cortes J, Gnant M, Houssami N, Poortmans P, Ruddy K, Tsang J, Cardoso F (2019). Breast cancer. Nat Rev Dis Primers.

[3] Apostolou P, Fostira F (2013). Hereditary breast cancer: the era of new susceptibility genes. Biomed Res Int.

[4] Al-Ejeh F, Smart CE, Morrison BJ, Chenevix-Trench G, Lopez JA, Lakhani SR, Brown MP, Khanna KK (2011). Breast cancer stem cells: treatment resistance and therapeutic opportunities. Carcinogenesis.

[5] Velasco-Velazquez MA, Popov VM, Lisanti MP, Pestell RG (2011). The role of breast cancer stem cells in metastasis and therapeutic implications. Am J Pathol.

[6] Wu Y, Sarkissyan M, Vadgama JV (2016). Epithelial-mesenchymal transition and breast cancer. J Clin Med.

[7] Freire-de-Lima L (2014). Sweet and sour: the impact of differential glycosylation in cancer cells undergoing epithelial-mesenchymal transition. Front Oncol.

[8] Baum LG (2016). Sweet beginning for cancer stem cells. Proc Natl Acad Sci U S A.

[9] Zhou D, Henion TR, Jungalwala FB, Berger EG, Hennet T (2000). The beta 1,3-galactosyltransferase beta 3GalT-V is a stage-specific embryonic antigen-3 (SSEA-3) synthase. J Biol Chem.

[10] Seko A, Kataoka F, Aoki D, Sakamoto M, Nakamura T, Hatae M, Yonezawa S, Yamashita K (2009). Beta1,3-galactosyltransferases-4/5 are novel tumor markers for gynecological cancers. Tumour Biol.

[11] Kuo HH, Lin RJ, Hung JT, Hsieh CB, Hung TH, Lo FY, Ho MY, Yeh CT, Huang YL, Yu J (2017). High expression FUT1 and B3GALT5 is an independent predictor of postoperative recurrence and survival in hepatocellular carcinoma. Sci Rep.

[12] Kolbl AC, Andergassen U, Jeschke U (2015). The role of glycosylation in breast cancer metastasis and cancer control. Front Oncol.

[13] Cazet A, Julien S, Bobowski M, Burchell J, Delannoy P (2010). Tumour-associated carbohydrate antigens in breast cancer. Breast Cancer Res.

[14] Chang WW, Lee CH, Lee P, Lin J, Hsu CW, Hung JT, Lin JJ, Yu JC, Shao LE, Yu J (2008). Expression of Globo H and SSEA3 in breast cancer stem cells and the involvement of fucosyl transferases 1 and 2 in Globo H synthesis. Proc Natl Acad Sci U S A.

[15] Cheung SK, Chuang PK, Huang HW, Hwang-Verslues WW, Cho CH, Yang WB, Shen CN, Hsiao M, Hsu TL, Chang CF (2016). Stage-specific embryonic antigen-3 (SSEA-3) and beta3GalT5 are cancer specific and significant markers for breast cancer stem cells. Proc Natl Acad Sci U S A.

[16] Chang WW, Lin RJ, Yu J, Chang WY, Fu CH, Lai A, Yu JC, Yu AL (2013). The expression and significance of insulin-like growth factor-1 receptor and its pathway on breast cancer stem/progenitors. Breast Cancer Res.

[17] Krebs AM, Mitschke J, Lasierra Losada M, Schmalhofer O, Boerries M, Busch H, Boettcher M, Mougiakakos D, Reichardt W, Bronsert P (2017). The EMT-activator Zeb1 is a key factor for cell plasticity and promotes metastasis in pancreatic cancer. Nat Cell Biol.

[18] Akrida I, Nikou S, Gyftopoulos K, Argentou M, Kounelis S, Zolota V, Bravou V, Papadaki H (2018). Expression of EMT inducers integrin-linked kinase (ILK) and ZEB1 in phyllodes breast tumors is associated with aggressive phenotype. Histol Histopathol.

[19] Sanchez-Tillo E, de Barrios O, Siles L, Cuatrecasas M, Castells A (2011). Postigo a: beta-catenin/TCF4 complex induces the epithelial-to-mesenchymal transition (EMT)-activator ZEB1 to regulate tumor invasiveness. Proc Natl Acad Sci U S A.

[20] Al-Hajj M, Wicha MS, Benito-Hernandez A, Morrison SJ, Clarke MF (2003). Prospective identification of tumorigenic breast cancer cells. Proc Natl Acad Sci U S A.

[21] Visvader JE, Lindeman GJ (2008). Cancer stem cells in solid tumours: accumulating evidence and unresolved questions. Nat Rev Cancer.

[22] Chuang PK, Hsiao M, Hsu TL, Chang CF, Wu CY, Chen BR, Huang HW, Liao KS, Chen CC, Chen CL (2019). Signaling pathway of globo-series glycosphingolipids and beta1,3-galactosyltransferase V (beta3GalT5) in breast cancer. Proc Natl Acad Sci U S A.

[23] Markopoulos C, Hyams DM, Gomez HL, Harries M, Nakamura S, Traina T, Katz A (2020). Multigene assays in early breast cancer: Insights from recent phase 3 studies. Eur J Surg Oncol.

[24] Caputo R, Cianniello D, Giordano A, Piezzo M, Riemma M, Trovo M, Berretta M, De Laurentiis M (2020). Gene expression assay in the management of early breast cancer. Curr Med Chem.

[25] Slodkowska EA, Ross JS (2009). MammaPrint 70-gene signature: another milestone in personalized medical care for breast cancer patients. Expert Rev Mol Diagn.

[26] Andre F, Ismaila N, Henry NL, Somerfield MR, Bast RC, Barlow W, Collyar DE, Hammond ME, Kuderer NM, Liu MC (2019). Use of biomarkers to guide decisions on adjuvant systemic therapy for women with early-stage invasive breast cancer: ASCO clinical practice guideline update-integration of results from TAILORx. J Clin Oncol.

[27] Zhang P, Sun Y, Ma L (2015). ZEB1: at the crossroads of epithelial-mesenchymal transition, metastasis and therapy resistance. Cell Cycle.

[28] Caramel J, Ligier M, Puisieux A (2018). Pleiotropic roles for ZEB1 in cancer. Cancer Res.

[29] Llorens MC, Rossi FA, Garcia IA, Cooke M, Abba MC, Lopez-Haber C, Barrio-Real L, Vaglienti MV, Rossi M, Bocco JL (2019). PKCalpha modulates epithelial-to-mesenchymal transition and invasiveness of breast cancer cells through ZEB1. Front Oncol.

[30] Hugo HJ, Pereira L, Suryadinata R, Drabsch Y, Gonda TJ, Gunasinghe NP, Pinto C, Soo ET, van Denderen BJ, Hill P (2013). Direct repression of MYB by ZEB1 suppresses proliferation and epithelial gene expression during epithelial-to-mesenchymal transition of breast cancer cells. Breast Cancer Res.

[31] Larsen JE, Nathan V, Osborne JK, Farrow RK, Deb D, Sullivan JP, Dospoy PD, Augustyn A, Hight SK, Sato M (2016). ZEB1 drives epithelial-to-mesenchymal transition in lung cancer. J Clin Invest.

[32] Ang L, Zheng L, Wang J, Huang J, Hu HG, Zou Q, Zhao Y, Liu QM, Zhao M, Wu ZS (2017). Expression of and correlation between BCL6 and ZEB family members in patients with breast cancer. Exp Ther Med.

[33] Chaffer CL, Marjanovic ND, Lee T, Bell G, Kleer CG, Reinhardt F, D'Alessio AC, Young RA, Weinberg RA (2013). Poised chromatin at the ZEB1 promoter enables breast cancer cell plasticity and enhances tumorigenicity. Cell.

[34] Holgersson J, Lofling J (2006). Glycosyltransferases involved in type 1 chain and Lewis antigen biosynthesis exhibit glycan and core chain specificity. Glycobiology.

[35] Hayashi N, Nakamori S, Okami J, Nagano H, Dono K, Umeshita K, Sakon M, Narimatsu H, Monden M (2004). Association between expression levels of CA 19-9 and N-acetylglucosamine-beta;1,3-galactosyltransferase 5 gene in human pancreatic cancer tissue. Pathobiology.

[36] Aronica A, Avagliano L, Caretti A, Tosi D, Bulfamante GP, Trinchera M (2017). Unexpected distribution of CA19.9 and other type 1 chain Lewis antigens in normal and cancer tissues of colon and pancreas: Importance of the detection method and role of glycosyltransferase regulation. Biochim Biophys Acta Gen Subj.

[37] Lin CH, Fan YY, Chen YY, Wang SH, Chen CI, Yu LC, Khoo KH (2009). Enhanced expression of beta 3-galactosyltransferase 5 activity is sufficient to induce in vivo synthesis of extended type 1 chains on lactosylceramides of selected human colonic carcinoma cell lines. Glycobiology.

[38] Magi-Galluzzi C, Maddala T, Falzarano SM, Cherbavaz DB, Zhang N, Knezevic D, Febbo PG, Lee M, Lawrence HJ, Klein EA (2016). Gene expression in normal-appearing tissue adjacent to prostate cancers are predictive of clinical outcome: evidence for a biologically meaningful field effect. Oncotarget.

[39] Slaughter DP, Southwick HW, Smejkal W (1953). Field cancerization in oral stratified squamous epithelium; clinical implications of multicentric origin. Cancer.

[40] Aran D, Camarda R, Odegaard J, Paik H, Oskotsky B, Krings G, Goga A, Sirota M, Butte AJ (2017). Comprehensive analysis of normal adjacent to tumor transcriptomes. Nat Commun.

