# Supplementary material for: High B3GALT5 expression confers poor clinical outcome and contributes to tumor progression and metastasis in breast cancer
Source: Breast Cancer Res. 2021 Jan 7;23:5. doi: 10.1186/s13058-020-01381-9 (PMC7792347; doi:10.1186/s13058-020-01381-9)
Supplement: Supplementary file 1 — Additional file 1: Figure S1. Clinical and pathological characteristics of 202 breast cancer patients. Figure S2. Correlation between B3GATL5 expression levels and stage or grade of breast cancers. Tumor (a and b) or adjacent non-tumor (c and d) tissues from patients with grade I, II, and III (a and c) or stage I, II, and III-IV (b and d) were analyzed for expression of B3GALT5 mRNA by using qRT-PCR. Statistical analysis was performed One-way ANOVA analysis. Figure S3. Expression levels of B3GALT5 in tumor of breast cancer tissues. The GSE1456 dataset was used to plot the survival curve comparing the patient with high (black) and low (gray) expression of B3GALT5. Figure S4. Higher expression of B3GALT5 in breast cancer tissue correlates with poor clinical outcome (RFS). Kaplan-Meier plots of relapse-free survival (RFS) comparing the 202 breast cancer patients with high (black) and low (gray) expression of B3GALT5 in tumor (a-c) and adjacent non-tumor parts (d-f) of patients with stage I-II (a and d), and stage I-II & Luminal A (b and e), and stage I-II & Luminal B (c and f) breast cancer were analyzed. Figure S5. Higher expression of B3GALT5 in breast cancer tissue correlates with poor clinical outcome (OS). Kaplan-Meier plots of overall survival (OS) comparing the 202 breast cancer patients with high (black) and low (gray) expression of B3GALT5 in tumor (a-c) and adjacent non-tumor parts (d-f) of patients with stage I-II (a and d), and stage I-II & Luminal A (b and e), and stage I-II & Luminal B (c and f) breast cancer were analyzed. Figure S6. Kaplan-Meier analyses of TNBC patients. Kaplan-Meier plots of RFS (a and c) or OS (b and d) comparing the TNBC breast cancer patients with high (black) and low (gray) expression of B3GALT5 in tumor (a and b) and adjacent non-tumor parts (c and d) were analyzed. Figure S7. Knockdown of B3GALT5 inhibits mammosphere formation. AS-B634 cells transfected with control siRNA (si-Ctrl) or B3GALT5 siRNA (si-B3GALT5-1 and -2) and the [file 13058_2020_1381_MOESM1_ESM.zip › 6 Revised Supplementary Figure Legends_Oct22.docx]

Supplementary Figure legends

**Fig S1. Clinical and pathological characteristics of 202 breast cancer patients.**

**Fig S2. Correlation between *B3GATL5* expression levels and stage or grade of breast cancers.** Tumor (a and b) or adjacent non-tumor (c and d) tissues from patients with grade I, II, and III (a and c) or stage I, II, and III-IV (b and d) were analyzed for expression of *B3GALT5* mRNA expression by using qRT-PCR. Statistical analysis was performed One-way ANOVA analysis.

**Fig S3. Expression levels of *B3GALT5* in tumor of breast cancer tissues.** The GSE1456 dataset was used to plot the survival curve comparing the patient with high (black) and low (gray) expression of B3GALT5.

**Fig S4. Higher expression of *B3GALT5* in breast cancer tissue correlates with poor clinical outcome (RFS).** Kaplan-Meier plots of relapse-free survival (RFS) comparing the 202 breast cancer patients with high (black) and low (gray) expression of *B3GALT5* in tumor (a-c) and adjacent non-tumor parts (d-f) of patients with stage I-II (a and d), and stage I-II & Luminal A (b and e), and stage I-II & Luminal B (c and f) breast cancer were analyzed.

**Fig S5. Higher expression of *B3GALT5* in breast cancer tissue correlates with poor clinical outcome (OS).** Kaplan-Meier plots of overall survival (OS) comparing the 202 breast cancer patients with high (black) and low (gray) expression of *B3GALT5* in tumor (a-c) and adjacent non-tumor parts (d-f) of patients with stage I-II (a and d), and stage I-II & Luminal A (b and e), and stage I-II & Luminal B (c and f) breast cancer were analyzed.

**Fig S6. Kaplan-Meier analyses of TNBC patients.** Kaplan-Meier plots of RFS (a and c) or OS (b and d) comparing the TNBC breast cancer patients with high (black) and low (gray) expression of *B3GALT5* in tumor (a and b) and adjacent non-tumor parts (c and d) were analyzed.

**Fig S7. Knockdown of *B3GALT5* inhibits mammosphere formation.** AS-B634 cells transfected with control siRNA (si-Ctrl) or B3GALT5 siRNA (si-B3GALT-1 and -2) and the representative pictures of mammosphere were shown. Scale bar: 250 µm

**Fig S8. Quantification of lung metastasis.** Lung sections were stained with H&E. Counting of the pre-metastatic colonies were based on the cell numbers per colony. Representative pictures were shown. Scale bar: 100 µm
